# Developing Conductive Highly Ordered Zinc Oxide Nanorods by Acetylacetonate-Assisted Growth

**DOI:** 10.3390/ma13051136

**Published:** 2020-03-04

**Authors:** Siti Shafura A Karim, Yuzuru Takamura, Phan Trong Tue, Nguyen Thanh Tung, Jamal Kazmi, Chang Fu Dee, Burhanuddin Yeop Majlis, Mohd Ambri Mohamed

**Affiliations:** 1 Institute of Microengineering and Nanoelectronics (IMEN), Universiti Kebangsaan Malaysia (UKM), 43600 Bangi, Selangor, Malaysia; shafura@ymail.com (S.S.A.K.); jamal_physics@outlook.com (J.K.); cfdee@ukm.edu.my (C.F.D.); burhan@ukm.edu.my (B.Y.M.); 2 School of Materials Science, Japan Advanced Institute of Science and Technology (JAIST), 1-1 Asahidai, Nomi City, Ishikawa 923-1292, Japan; takamura@jaist.ac.jp; 3 Laboratory for Materials and Structures, Department of Materials Science and Engineering, Tokyo Institute of Technology (TITech), Yokohama 226-8503, Japan; phan.t.ac@m.titech.ac.jp; 4 Department of Finemechanics, Graduate School of Engineering, Tohoku University, 6-6, Aramaki Aza Aoba, Aoba-ku, Sendai 980-8579, Japan; nguyen.thanh.tung@hane.mech.tohoku.ac.jp

**Keywords:** acetylacetonate, conductive, highly ordered, hydrothermal, nanorods, vertical growth, zinc oxide

## Abstract

Highly ordered vertically grown zinc oxide nanorods (ZnO NRs) were synthesized on ZnO-coated SiO_2_/Si substrate using zinc acetylacetonate hydrate as a precursor via a simple hydrothermal method at 85 °C. We used 0.05 M of ZnO solution to facilitate the growth of ZnO NRs and the immersion time was varied from 0.5 to 4 h. The atomic force microscopy revealed the surface roughness of ZnO seed layer used to grow the ZnO NRs. The morphology of vertically grown ZnO NRs was observed by field emission scanning electron microscopy. X-ray diffraction examination and transmission electron microscopy confirmed that the structure of highly ordered ZnO NRs was crystalline with a strong (002) peak corresponded to ZnO hexagonal wurtzite structure. The growth of highly ordered ZnO NRs was favorable due to the continuous supply of Zn^2+^ ions and chelating agents properties obtained from the acetylacetonate-derived precursor during the synthesis. Two-point probe current–voltage measurement and UV–vis spectroscopy of the ZnO NRs indicated a resistivity and optical bandgap value of 0.44 Ω.cm and 3.35 eV, respectively. The photoluminescence spectrum showed a broad peak centered at 623 nm in the visible region corresponded to the oxygen vacancies from the ZnO NRs. This study demonstrates that acetylacetonate-derived precursors can be used for the production of ZnO NRs-based devices with a potential application in biosensors.

## 1. Introduction

Zinc oxide (ZnO) is a versatile n-type semiconducting material owing to the direct bandgap (3.37 eV) with a large exciton binding energy (60 meV) at room temperature, bio-safe, functional biocompatible, and high isoelectric point (~9.5) [1,2]. Among all diverse morphologies of ZnO nanostructures such as nanosheets, nanoflakes, nanoplates, nanoflowers, and nanocombs [3,4,5,6,7,8,9,10], nanorods (NRs) have higher surface area and catalytic properties, and for that reason, they can be utilized in many potential applications such as sensors, field-effect transistors, piezoelectric generators, and biosensors [5,7,11,12]. Highly ordered ZnO NRs with excellent electrical conductivity give higher surface reaction activity for a bio-interfacing platform for immobilization, which could lead to greater signal transductions during the detection, hence, promise better sensing performance. For instance, N.S. Ridhuan et al. reported that the highly oriented ZnO NRs with an aspect ratio of ~6 showed a good matrix for glucose oxidase immobilization and obtained a high sensitivity of 48.75 µA/mM·cm^2^ towards glucose detection [13]. Therefore, ZnO NRs have been explores as a promising biomaterial for biosensor development. Several methods have been developed to synthesize ZnO nanostructures, such as pulsed laser deposition (PLD), physical vapor deposition (PVD), chemical vapor deposition (CVD), carbothermal reduction method, and hydrothermal method [14,15,16,17,18,19]. However, these methods (such as PLD, PVD and CVD) require a high vacuum chamber and more complex set up to deposit ZnO thin film. The hydrothermal method offers simplest preparation set-up with less energy consumption, and low production cost, where the ZnO nanostructures can be grown under mild synthesis conditions (such as normal pressure and low growth temperature), simple facility, good repeatability and high reliability [20,21]. In hydrothermal method, precursor has a role as a Zn^2+^ ions source. There are many reports on the preparation of ZnO NRs using nitrate and acetate-derived precursor, however, they only able to obtain low aspect ratio nanorods and very time consuming [2,13,22,23,24,25]. Few works have demonstrated the development of precipitated ZnO prepared by acetylacetonate-derived precursor via a chemical method, such as hydrolysis method [26,27], and solvothermal method [28]. However, no works reported on the growth of ZnO NRs directly on a seeded substrate using the acetylacetonate-derived precursor. ZnO NRs that grown directly on the substrate provides good adherence and stability for enhanced device performance [11,13].

Therefore, we developed a simple hydrothermal process to synthesize ZnO NRs using zinc acetylacetonate hydrate as a precursor. To the best of our knowledge, this is the first report on the synthesis of ZnO NRs using acetylacetonate-assisted hydrothermal method on ZnO-coated SiO_2_/Si substrate. The selection of acetylacetonate-derived as an alternative precursor for ZnO growth because they are inexpensive, non-toxic, and commercially available. Furthermore, the precursor is less susceptible to hydrolysis as compared to halide-derived or acetate-derived; which is due to their chelating agent properties [29,30]. The chelating agent properties provide stable multidentate ligand to the non-polar facets of ZnO crystal, hence favor the growth in the single polar (001) facets. These properties are believed can influence the growth of ZnO. The quality of ZnO NRs synthesized using this approach has been systematically studied in terms of their morphological, structural, optical, and electrical properties. In addition, a possible growth mechanism of the ZnO NRs has been proposed.

## 2. Materials and Methods

Zinc acetate dehydrate, 2-methoxyethanol and monoethanolamine were used as a starting material to prepare a ZnO seed layer solution. The 0.2 M of ZnO solution was stirred at 110 °C for 30 min. A clear and homogeneous solution was obtained, and spin-coated onto cleaned SiO_2_/Si substrate (2 × 2 cm^2^) at 2000 rpm for 30 s. Then, the ZnO-coated SiO_2_/Si substrate was annealed at 350 °C for 30 min. For ZnO NRs preparation, equimolar 0.05 M ZnO solution was prepared by dissolving 0.198 g of zinc acetylacetonate hydrate (Zn(AcAc)_2_, Zn(C_5_H_7_O_2_)_2_·xH_2_O), and 0.084 g of hexamethylenetetramine (HMTA, (CH_2_)_6_N_4_)) in 15 mL of deionized water. Next, the solution was sonicated at 50 °C for 30 min, and then stirred at room temperature for 3 h. The solution was poured into a capped bottle, and the ZnO-coated SiO_2_/Si substrate was immersed in the solution. The immersion process was carried out at 85 °C with the immersion time varied between 0.5 to 4 h. Next, the sample was rinsed with deionized water and dried in an oven at 150 °C for 30 min. Finally, the sample was annealed at 350 °C for 30 min in N_2_ ambient.

The surface roughness of the ZnO seed layer was measured using atomic force microscopy (Hitachi, AFM5000II). The morphological and cross-sectional view of the ZnO NRs were observed by field emission scanning electron microscopy (FESEM, Zeiss Merlin), and transmission electron microscopy (TEM, Thermo Scientific, Talos L120C, 120 kV). Meanwhile, the structural properties of the ZnO NRs were characterized using thin-film X-ray diffraction (XRD, PANalytical, X’Pert Pro, CuKα). The optical properties of ZnO NRs were characterized by a diffuse reflectance method over the range of 300–800 nm using a Hitachi U39000-H spectrophotometry system. In addition, the photoluminescence properties of ZnO NRs were characterized using the 325 nm line of a He–Cd UV laser (Renishaw InVia) as the excitation source at room temperature with laser power of 5 mW. For electrical characterization purposes, metal contact electrodes consisting of Au (40 nm)/Cr (10 nm) were deposited by thermal evaporator and electron beam evaporator, respectively. The distance between the two electrodes was approximately 20 µm. The electrical properties of ZnO NRs were measured using the current-voltage (I–V) semiconductor characterization system (Keithley 4200-SCS).

## 3. Results and Discussion

### 3.1. Morphological Properties of Zinc Oxide Nanorods (ZnO NRs)

**Figure 1:**
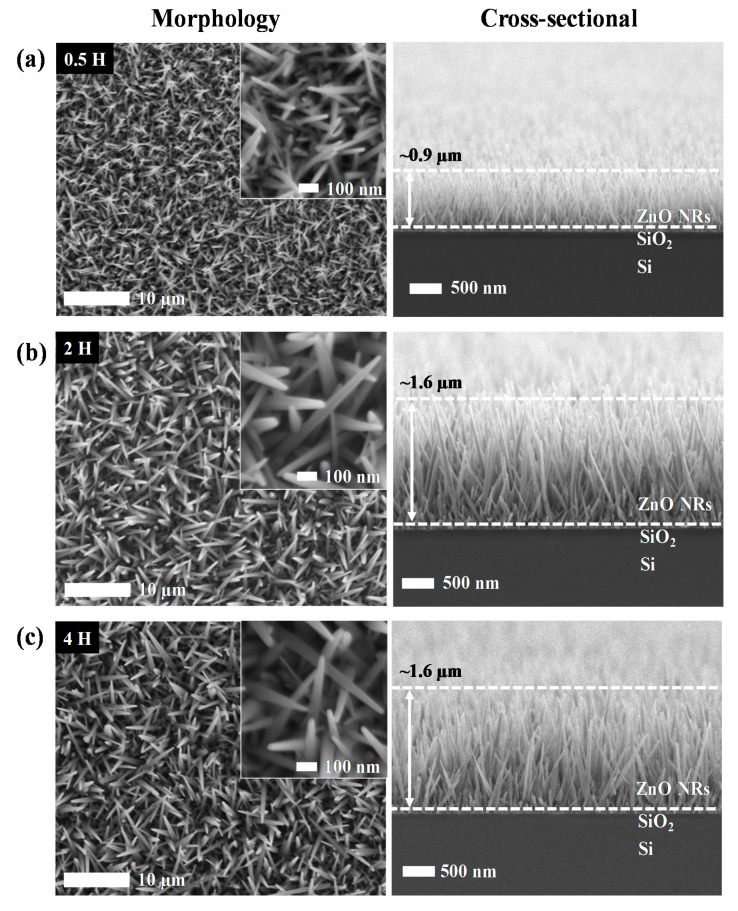
Field emission scanning electron microscopy (FESEM) images of the ZnO NRs after immersion time of (**a**) 0.5 h, (**b**) 2 h, and (**c**) 4 h with different magnification in between their morphological and cross-sectional images.

Figure 1 shows the morphological and cross-sectional FESEM images of ZnO NRs obtained at different immersion time between 0.5 h to 4 h. All samples were observed to have high uniformity and well-distribution of ZnO NRs on the surface of the substrate. Interestingly, the increment of immersion time resulted in a higher density of grown ZnO NRs, thus developed more interconnection between them. The diameter of ZnO NRs for 0.5 h is distributed in the range of 15–30 nm with an average value of 25 nm. As the immersion time was increased to 2 h, the diameter of ZnO NRs increased to the range of 30–60 nm with an average value of 40 nm. Apparently, a similar diameter range of ZnO NRs was obtained after further increment of immersion time to 4 h. From the cross-sectional FESEM images (Figure 1), it was observed that the growth of ZnO NRs is most likely vertical to the substrate with uniform length. The FESEM image of 0.5 h sample (Figure 1a) showed the length of the ZnO NRs were approximately 0.9 µm. As the immersion time increased to 2 h, the length of the ZnO NRs were approximately 1.6 µm which is almost twice longer than 0.5 h sample, which is an improvement with respect to other hydrothermal method that used acetates and nitrates-derived precursor to grow nanorods (the minimal immersion time captured from several works to grow nanorods are presented in Table 1). However, a further increment in the immersion time to 4 h showed insignificant differences in terms of the morphologies and dimensionality of the ZnO NRs, which can be ascribed to the insufficient amount of Zn^2+^ ions supplied to the system, hence abrupted the growth of ZnO NRs [31]. These showed that in a hydrothermal, precursor plays a role in supplying Zn^2+^ ions to the system in order to completely form ZnO. Table 2 shows the list of diameter and length of ZnO NRs obtained at different immersion time.

**Figure 2:**
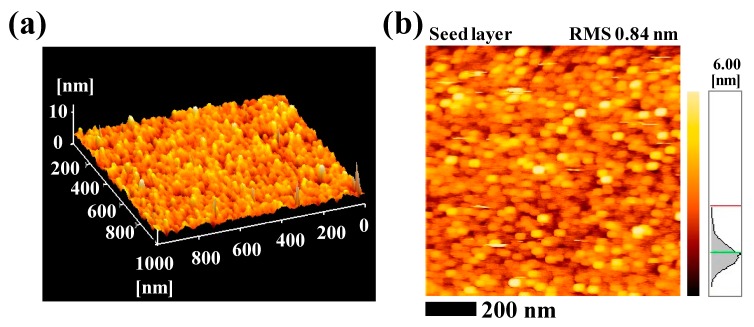
(**a**) 3D atomic force microscopy (AFM) image of ZnO-coated SiO_2_/Si substrate deposited using the spin coating technique, and (**b**) the surface roughness.

The morphologies of grown ZnO NRs are mainly attributed to the surface roughness of the seed layer. Figure 2 shows the AFM topographic images of the ZnO seed layer that provides nucleation sites for the growth of the ZnO NRs. The ZnO seed layer is uniformly distributed at the entire surface as shown in 3D AFM images (Figure 2a) with the roughness value of 0.84 nm (Figure 2b). This uniform distribution of nucleation sites contributed to the alignment and density of the grown ZnO NRs.

### 3.2. Structural Properties of ZnO NRs

**Figure 3:**
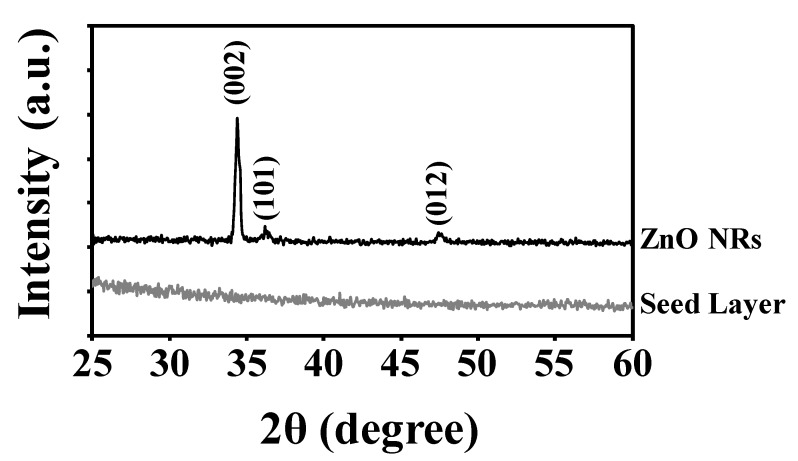
X-ray diffraction (XRD) patterns of ZnO NRs and ZnO seed layer.

The crystal structure of ZnO NRs prepared for 2 h was confirmed using XRD analysis. All peaks were observed at 34.38°, 36.18° and 47.46° of 2θ in the ZnO NRs as shown in Figure 3, which corresponds to the (002), (101) and (012) diffraction peaks of hexagonal wurtzite for ZnO crystal family (PDF code no: 01-079-0207), respectively. The sharp and strong peak (002) indicates the growth of ZnO NRs were dominantly along the *c*-axis direction without any impurities. The wurtzite lattice parameters, such as the values of *d* and the distances between adjacent crystal planes (*hkl*), were calculated using the Bragg formula,

*λ* = 2*d* sin θ. (1)

The (002), (101) and (012) planes exhibited *d*-spacing of 0.2606, 0.2481 and 0.1914 nm, respectively. Values obtained were found to be consistent as reported in the literature [32]. Meanwhile, the well-known Scherrer formula [33],

*D* = (*kλ*/*β cos θ*), (2)

was used to determine the crystallite size of the ZnO NRs where *D* is the crystallite size in nanometers (nm), λ is the wavelength of the radiation (1.54056 Å for CuK_α_ radiation), *k* is the constant that equal to 0.94, *β* is the full width at half-maximum (FWHM) of peak intensity, and *θ* is the peak position. The (002) peak was chosen as the preferred peak to calculate the crystallite size. The crystallite size of ZnO NRs was calculated to be approximately 20 nm. The XRD results indicate our acetylacetonate-assisted growth produced crystalline ZnO NRs with preferential growth along c-axis.

**Figure 4:**
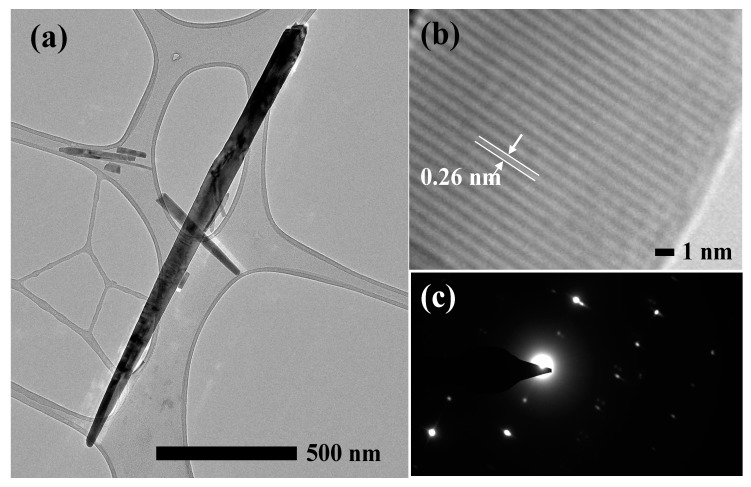
Transmission electron microscopy (TEM) images of ZnO NRs synthesized for 2 h at (**a**) low magnification TEM image, (**b**) high magnification TEM image, and (**c**) the selected area of electron diffraction (SAED) pattern, obtained at 120 kV.

The crystal structure of ZnO NRs synthesized in 2 h was further characterized using a 120 kV TEM. The sharp nano-grass structure was observed as presented in Figure 4a. The length and diameter of the nanorod were measured to be approximately 1.6 µm and 40 nm, respectively, which is consistent with the FESEM results in terms of their morphologies and dimensionality. Meanwhile, the high magnification TEM image (Figure 4b) reveals the distance between two parallel fringes, which is approximately 0.26 nm, and it is fully consistent with the XRD result in terms of the d-spacing of the (002) planes of the wurtzite hexagonal of ZnO crystal. It confirms that the obtained ZnO NRs are crystalline with the wurtzite hexagonal structure, and are grown along the c-axis orientation, which is further affirmed by the selected area of electron diffraction (SAED) pattern, as shown in Figure 4c.

### 3.3. Growth Mechanism of ZnO NRs

The ZnO NRs were grown by heating up the mixture of Zn(AcAc)_2_, HMTA, and deionized water at 85 °C. HMTA reacts with deionized water and decomposed to formaldehyde and ammonia as intermediate, as shown in Equation (3). Then the ammonia will further react with deionized water to produce ammonium ions (NH_4_^+^) and hydroxyl ions (OH^−^) as shown in Equation (4). This process is known as the hydrolyzation process.

(CH_2_)_6_N_4_ + 6H_2_O ↔ 6HCHO + 4NH_3_ (3)

NH_3_ + H_2_O ↔ NH_4_^+^ + OH^−^. (4)

Concurrently, the hydrolyzation process and Equation (5) also occurred [34]. As a result, Zn^2+^ ions and acetylacetonate ions are continuously supplied to the system.

Zn(CH_3_COCHCOCH_3_)_2_.H_2_O → Zn^2+^ + (CH_3_COCHCOCH_3_) + H_2_O^−^. (5)

As the concentration of Zn^2+^ and OH^−^ ions achieve a stable state at minimum energy level, the ZnO starts to grow rapidly to form rod-like structures. The formation of crystalline ZnO from Zn(OH)_2_ can be explained by these simple chemical Equations:

Zn^2+^ + 2OH + 2H_2_O → Zn(OH)_2_ + 2H_2_O ↔ ZnO + H_2_O. (6)

**Figure 5:**
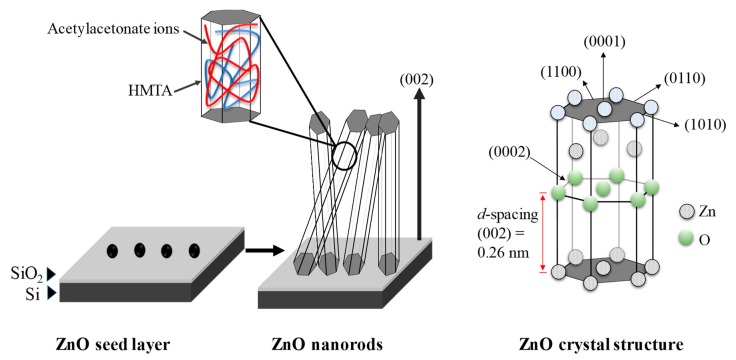
Schematic illustration of the possible growth mechanism of the ZnO NRs synthesized using the acetylacetonates-assisted hydrothermal method.

The growth of ZnO NRs is involved by the energetic and kinetic factor. After reaching the critical value, the ZnO starts to grow rapidly to form sharp nano-grass structures as can be seen in FESEM and TEM results. Acetylacetonate-derived precursor is soluble in HMTA, and both exhibit non-polar chelating agent properties [29,35,36,37]. They form stable multidentate ligand with the (101) non-polar facets of ZnO crystal, therefore, encouraging the growth in the single polar (001) facets. The velocity in the (0001) direction or c-axis is the fastest [38], hence ZnO NRs growth occurs mostly along the *c*-axis. The XRD and TEM results confirmed that the grown ZnO NRs have crystalline wurtzite structure along *c*-axis as the preferred orientation. From literatures, B. Ruqia et al. demonstrated that the growth of ZnO NRs along the (0001) direction by a direct solvothermal treatment was affected by controlling the concentration of the precursor and capping agents [28]. At lower concentrations of the precursor, they obtained an inhomogeneous low aspect ratio of nanorods, however, the aspect ratio of nanorods was increased with the use of octylamine as a capping agent. Meanwhile, Y. Chen et al. reported the high sputtering pressure was found to disturb the growth of the columnar structure of the ZnO deposited by radio frequency magnetron sputtering [39]. The illustration of the possible growth mechanism for the highly ordered ZnO NRs synthesized on the ZnO-coated SiO_2_/Si substrate using the acetylacetonate-assisted hydrothermal method are presented in Figure 5.

### 3.4. Electrical Properties of ZnO NRs

**Figure 6:**
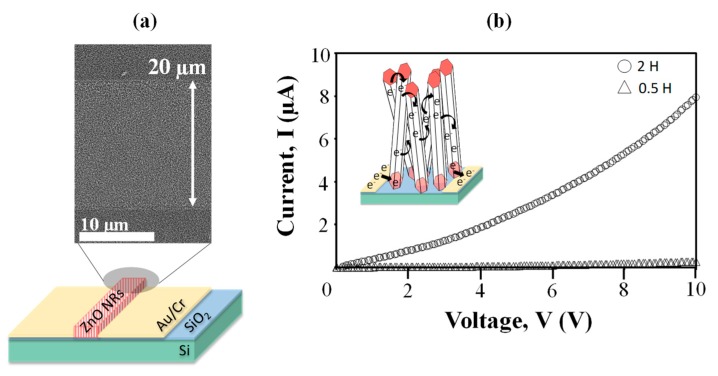
(**a**) Metal-semiconductor-metal structure used to measure electrical properties of the ZnO NRs, with its morphological FESEM image. (**b**) I–V curve of the ZnO NRs at different immersion time of 0.5 h and 2 h. Inset shows an illustration of electron movement in ZnO NRs.

For electrical conductivity studies, the ZnO NRs were deposited on a fabricated channel with a distance of 20 µm, as showed in Figure 6a. The bending curve indicated a semiconducting behavior of the ZnO NRs, as can be seen from Figure 6b. As the voltage was swept from 0 to 10 V, 2 h sample shows higher current flow compared to 0.5 h sample. Higher density of ZnO NRs produced more interconnection between the nanorods, hence, induced more electron pathway, as illustrated in inset Figure 6b. The resistivity of the ZnO NRs can be calculated using this formula (7) [40], resistivity;

*ρ* = (*V/I*)(*wt/l*), (7)

where V is the applied voltage, I is the measured current, w is the length of metal contact, *t* is the thickness of the sample, and l is the length of the channel. It shows that the 2 h sample exhibited a lower resistivity value of 0.44 Ω.cm, compared to a 0.5 h sample (7.16 Ω.cm). From literature, the electrical resistivity of ZnO was reported to be in the range of 10^−2^ to 10 Ω.cm [41,42].

### 3.5. Optical Properties of ZnO NRs

**Figure 7:**
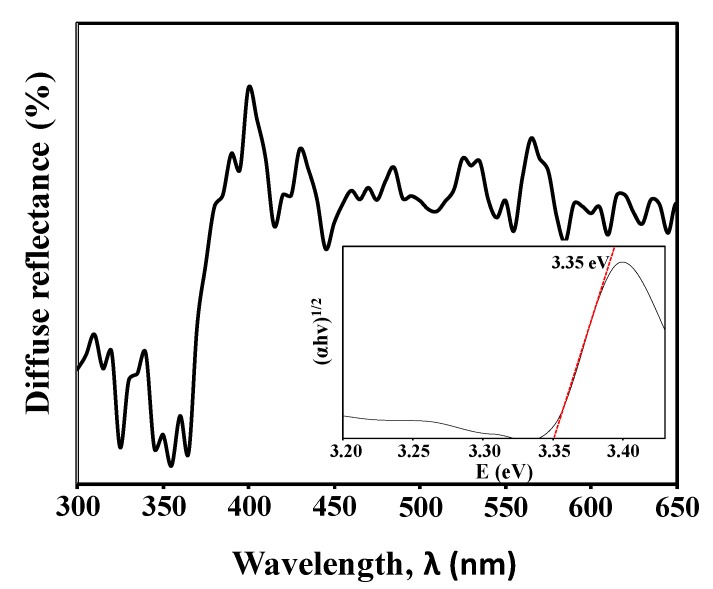
The UV–vis diffuse reflectance spectrum of ZnO NRs obtained at an immersion time of 2 h. Inset shows the Tauc plot of the spectrum.

The room temperature UV-vis diffuse reflectance spectrum of ZnO NRs obtained after 2 h of immersion time is shown in Figure 7. The spectrum reveals a rapid change in the diffuse reflectance of the ZnO NRs at a wavelength of 370 nm which can be explained to the reflection of the intrinsic bandgap of ZnO due to the electron transitions from the valence band to the conduction band (O_2p_→Zn_3d_) [43]. Tauc’s plot was used to determine the optical bandgap energy of the ZnO NRs by drawing the tangent line at the lower energy sides. The Tauc’s plot of the diffuse reflectance of the ZnO NRs is presented in the inset of Figure 7, and it exhibits a bandgap of 3.35 eV. These electrical and optical properties of ZnO NRs prove its applicability in biosensor applications [13,44]. This is due to the fact that ZnO NRs could provide higher surface reaction activity for biomolecules immobilization, and their good electrical conductivity could enhance signal transductions during the detection, hence resulting in greater device performances.

**Figure 8:**
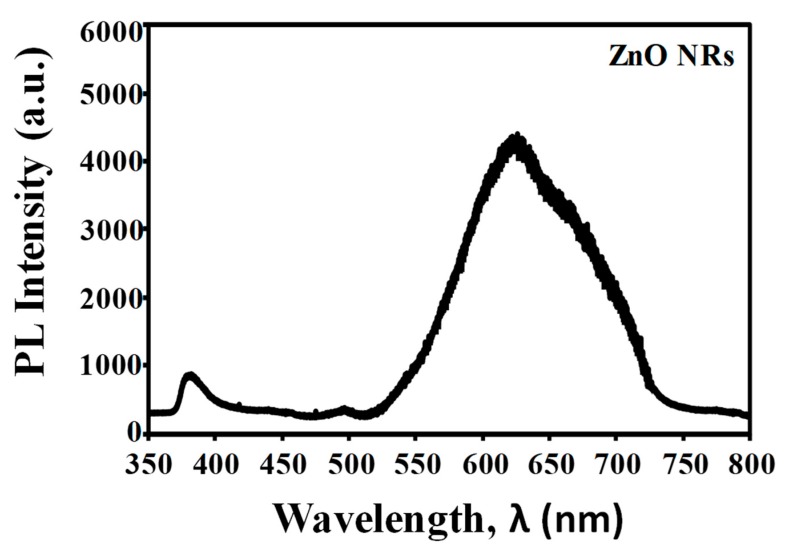
The PL spectrum of ZnO NRs obtained at an immersion time of 2 h.

The room temperature photoluminescence (PL) properties of ZnO NRs obtained after 2 h of immersion time in the range of 350–800 nm are shown in Figure 8. The PL spectrum of the ZnO NRs shows two peaks centered at 383 nm and 623 nm, which corresponded to the ultraviolet emission peak and the green emission peak, respectively. The ultraviolet emission is also known as near band edge emission (NBE), attributed by the free excitons recombination through excitons collision process [28,45]. The visible emission of ZnO NRs appeared as a broad peak centered at 623 nm with a bandwidth of 250 nm. This emission peak is related to the presence of oxygen vacancies in the ZnO crystals [46]. These defect structures in ZnO crystal will enhance the surface defect activities, thus allowing them to provide strong binding sites for the absorption of various organic and inorganic molecules [47,48]. Consequently, this may improve the biomolecules immobilization for future biosensing devices.

## 4. Conclusions

Highly ordered vertically grown ZnO NRs have been successfully synthesized using a zinc acetylacetonate-assisted hydrothermal method on a ZnO-coated SiO_2_/Si substrate. A uniform, dense, and interconnected ZnO NRs were obtained after 2 h of immersion time. The synthesized ZnO NRs have an average length of 1.6 µm and an average diameter of 40 nm. XRD examination confirms the ZnO NRs are hexagonally crystalline with strong preferential growth at c-axis. TEM analysis further reveals the distance between two parallel fringes of ZnO NRs measured to be approximately 0.26 nm, which reflected the d-spacing of (002) plane. Chelating agent properties of acetylacetonate ions have influenced the growth of ZnO NRs. Furthermore, the electrical resistivity and optical bandgap energy of ZnO NRs were measured to be around 0.44 Ω.cm and 3.35 eV, respectively. A broad peak centered at 623 nm in the visible region was observed in photoluminiscense spectrum that corresponded to the oxygen vacancies from the ZnO NRs. With a good crystallinity, a highly ordered growth, a high active surface area, and a low electrical resistivity, it could be suggested that the synthesized ZnO NRs by this method is suitable to be used as a transducing element in biosensor applications.

## Figures and Tables

**Table 1:** Minimal immersion time to grow ZnO nanorods (NRs) on a seeded substrate using various types of precursors with their dimensionality of obtained nanorods.

No.	Precursor	Immersion Time (h)	Length of Nanorods (µm)	Diameter of Nanorods (nm)	Aspect Ratio (Length/Diameter)	Ref.
1	Nitrate	4	0.645	109.9	6	[13]
2	Nitrate	N/A ^1^	1.2	~230	5	[22]
3	Nitrate	4	2	~150	14	[23]
4	Nitrate	3	4	~150	27	[24]
5	Acetate	4	0.15–0.40	50-60	8	[2]
6	Acetate	6	4	N/A ^1^	-	[25]
7	Acetylacetonate	2	1.6	~40	40	this work

^1^ not reported.

**Table 2:** The summary of length and diameter of prepared ZnO NRs at different immersion time.

Immersion Time (h)	Length (µm)	Diameter (nm)	Aspect Ratio (Length/Diameter)
0.5	0.9	~25	36
2	1.6	~40	40
4	1.6	~40	40
